# Natural Products in Clear Cell Renal Cell Carcinoma: Rewiring the VHL-HIF Axis, Metabolic Plasticity, and Tumor–Immune Interactions

**DOI:** 10.3390/ijms27104584

**Published:** 2026-05-20

**Authors:** Yao-Chou Tsai, Chung-Che Tsai, Vincent F. S. Tsai, Chih-Hung Lin, Chan-Yen Kuo

**Affiliations:** 1 Division of Urology, Department of Surgery, Taipei Tzuchi Hospital, The Buddhist Tzu Chi Medical Foundation, New Taipei City 23142, Taiwan; tsai1970523@yahoo.com.tw (Y.-C.T.); ntubala@yahoo.com.tw (V.F.S.T.); 2 School of Medicine, Buddhist Tzu Chi University, Hualien 97004, Taiwan; 3 Department of Nursing, Cardinal Tien College of Healthcare and Management, New Taipei City 231038, Taiwan; chungche.tsai@gmail.com; 4 Department of Internal Medicine, Cathay General Hospital, Taipei 106438, Taiwan; 5 Institute of Oral Medicine and Materials, College of Medicine, Tzu Chi University, Hualien 97004, Taiwan

**Keywords:** ccRCC, VHL, HIF, tumor microenvironment, natural products

## Abstract

Clear cell renal cell carcinoma (ccRCC) is driven by von Hippel-Lindau (VHL) tumor suppressor loss and persistent activation of hypoxia-inducible factors (HIFs), which coordinately regulate angiogenesis, metabolic reprogramming, redox balance, and tumor–immune interactions. Although immune checkpoint inhibitors and vascular endothelial growth factor-targeted therapies have improved outcomes, resistance remains common due to adaptive network plasticity. Selected natural products have been reported to exhibit multitarget regulatory activities that may influence interconnected oncogenic pathways. This review highlights how compounds such as curcumin, resveratrol, quercetin, and epigallocatechin-3-gallate modulate the VHL-HIF axis, disrupt metabolic and redox homeostasis, and influence tumor–immune system interactions in ccRCC. We propose a system-level framework in which natural products enhance therapeutic sensitivity; however, further validation is required for clinical translation.

## 1. Introduction

Clear cell renal cell carcinoma (ccRCC) accounts for approximately 70–80% of all renal malignancies and remains a major cause of cancer-related mortality worldwide [1]. ccRCC occupies a unique position among solid tumors not only because of its high prevalence among kidney cancers but also because of its distinctive metabolic and vascular phenotypes [2]. Central to this phenotype is the loss of von Hippel-Lindau (VHL) function, which abolishes oxygen-dependent ubiquitination and proteasomal degradation of hypoxia-inducible factor (HIF)-α subunits, thereby enabling their constitutive accumulation and sustained transcriptional activation of angiogenic and metabolic gene networks [3]. The downstream consequences are multifaceted, involving augmented angiogenic signaling, increased glucose flux, and extensive metabolic reprogramming, including lipid accumulation and mitochondrial dysfunction, which collectively sustain the bioenergetic and anabolic requirements of tumor progression [4].

Recent studies position ccRCC as a metabolically rewired malignancy in which dysregulated glucose, lipid, and mitochondrial pathways are intricately linked to redox imbalance and immune modulation; collectively, these factors drive tumor progression, heterogeneity, and therapeutic resistance [5]. Despite substantial improvements in clinical outcomes with immune checkpoint blockade and anti-angiogenic therapies, numerous patients exhibit primary or acquired resistance, ultimately resulting in disease progression and highlighting the dynamic and adaptive crosstalk between tumor-intrinsic programs and the tumor microenvironment [6].

In this context, selected natural products are increasingly recognized as bioactive compounds capable of modulating multiple interconnected signaling pathways, thereby enabling broader regulation of oncogenic networks beyond single-pathway inhibition [7]. Such multitarget activity may be especially relevant in ccRCC, where therapeutic resistance arises from pathway redundancy, metabolic reprogramming, and dynamic crosstalk between intrinsic tumor signaling and microenvironment, enabling adaptive survival under therapeutic pressure [8]. Accordingly, this review proposes that ccRCC should be conceptualized as a system-level malignancy driven by a VHL loss-induced pseudohypoxic state that integrates metabolic rewiring, redox imbalance, and immune suppression into a self-reinforcing adaptive network. Within this framework, natural products may function as network-level modulators capable of partially perturbing the interconnected VHL-HIF-metabolism-immune axis, thereby exposing latent vulnerabilities and enhancing therapeutic responsiveness in VHL-deficient tumors.

**Figure 1:**
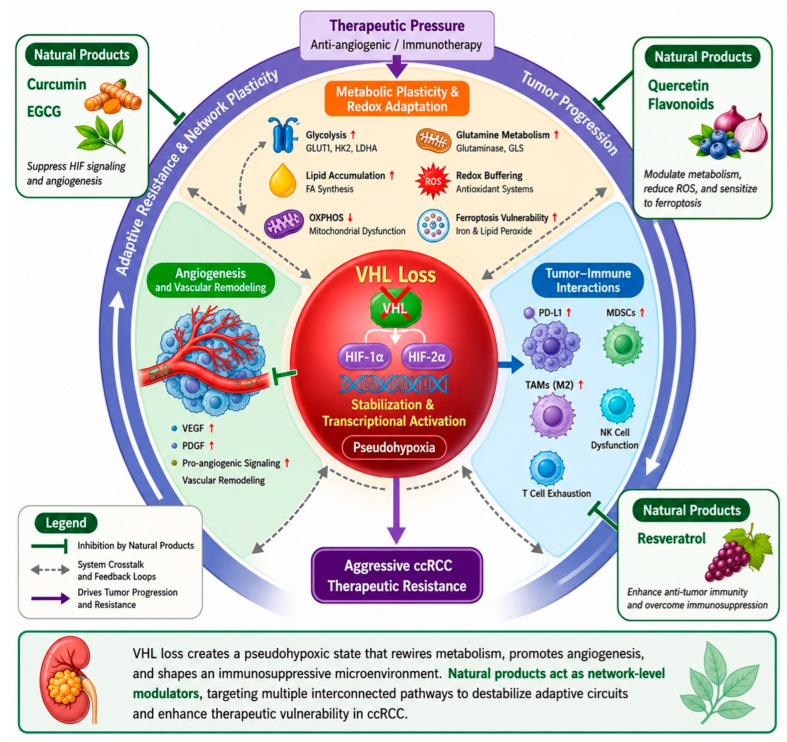
Systems-level architecture of von Hippel-Lindau (VHL)-deficient clear cell renal cell carcinoma and network-based intervention by natural products. Loss of VHL function stabilizes hypoxia-inducible factor (HIF)-1α and HIF-2α, establishing a pseudohypoxic state that serves as a central organizing hub in clear cell renal cell carcinoma (ccRCC). This program drives angiogenesis, metabolic reprogramming, redox adaptation, and immunosuppressive remodeling, collectively forming a dynamic network that promotes tumor progression and therapeutic resistance. Natural products, including curcumin, resveratrol, quercetin, and epigallocatechin-3-gallate (EGCG), may act as multi-target modulators by simultaneously perturbing these pathways. Through coordinated interference with HIF signaling, metabolism, and immune regulation, they may destabilize adaptive network resilience, expose vulnerabilities such as ferroptosis sensitivity, and enhance therapeutic responsiveness in ccRCC. This figure represents a conceptual framework summarizing currently proposed interactions and should not be interpreted as a quantitatively validated mechanistic model. The figure was generated with assistance from ChatGPT (OpenAI) version 5.2 and subsequently manually modified, annotated, and scientifically validated by the authors. ↑: Up-regulation; ↓: Down-regulation.

To conceptualize the system-level architecture of ccRCC, we propose an integrated model in which loss of the *VHL* tumor suppressor establishes a persistent pseudohypoxic state by stabilizing HIF-1α and HIF-2α. This central oncogenic hub drives angiogenesis, metabolic plasticity, redox adaptation, and tumor–immune remodeling, forming a highly interconnected and dynamically adaptive network that underlies tumor progression and therapeutic resistance. Within this framework, natural products are positioned as multitarget modulators that simultaneously perturb multiple nodes across these pathways. By attenuating angiogenic signaling, disrupting metabolic homeostasis, and partially reprogramming the immunosuppressive tumor microenvironment, these agents may destabilize network robustness and enhance therapeutic vulnerability in VHL-deficient tumors (Figure 1).

## 2. VHL Loss Drives Pseudohypoxic Tumor Progression in ccRCC

Loss of VHL function represents the initiating and defining molecular event in most ccRCCs; however, its biological impact extends far beyond canonical oxygen-sensing mechanisms [9]. In addition to its canonical role in regulating HIF stability through oxygen-dependent ubiquitination and proteasomal degradation, VHL serves as a substrate-recognition component of the E3 ubiquitin ligase complex that governs a broader spectrum of ubiquitin-mediated proteostasis. Beyond HIF-α, VHL targets multiple proteins involved in receptor signaling, transcriptional regulation, and cell cycle control, including epidermal growth factor receptor, atypical PKC, and RNA polymerase II subunits, thereby modulating signaling fidelity and cellular homeostasis [10]. Moreover, VHL exerts HIF-independent functions for maintaining chromosomal stability, regulating nuclear factor kappa B activity, and controlling ciliary dynamics, highlighting its role as a multifunctional tumor suppressor rather than as a mere oxygen sensor [10]. Disruption of this VHL-dependent ubiquitin regulatory network abrogates oxygen-dependent proteasomal degradation of HIF-α, leading to its pathological stabilization and sustained transcriptional activation of hypoxia-responsive genes [11]. Concurrently, VHL loss disrupts the turnover of additional ubiquitin substrates, leading to dysregulation of proteostasis and signaling. This convergence, along with constitutive HIF activation, establishes a permissive oncogenic landscape that promotes tumor progression and adaptive stress responses [9,12,13].

Stabilized HIF-1α and HIF-2α act as master transcriptional regulators that coordinate angiogenesis, metabolic reprogramming, and immune modulation, thereby orchestrating tumor progression and adaptation to hypoxic stress [14]. Mechanistically, HIF signaling drives the transcription of genes involved in vascular remodeling, including vascular endothelial growth factor (VEGF) and platelet-derived growth factor (PDGF), as well as glucose uptake and glycolysis, such as glucose transporter 1 (GLUT1) and hexokinase 2 (HK2), while regulating lipid metabolism and redox homeostasis, collectively establishing a metabolic phenotype optimized for tumor growth under fluctuating oxygen conditions [15,16,17,18]. Notably, this transcriptional rewiring extends to the tumor immune microenvironment, where HIF activity promotes the recruitment of immunosuppressive myeloid populations, enhances programmed death-ligand 1 (PD-L1) expression, and reshapes cytokine networks, thereby facilitating immune evasion [19].

In ccRCC, the sustained activation of HIF-dependent programs under VHL deficiency establishes a pseudohypoxic state that integrates angiogenesis and metabolic reprogramming with hypoxia-driven immunomodulation, thereby reprogramming the tumor microenvironment toward an immunosuppressive phenotype [20]. This state is not merely a passive consequence of oxygen deprivation, but rather an actively maintained oncogenic program driven by sustained HIF signaling, which promotes tumor progression, immune evasion, and metabolic adaptation. This state reinforces tumor heterogeneity and therapeutic resistance [21,22]. Emerging evidence further indicates that HIF-2α plays a dominant oncogenic role in sustaining proliferative signaling and tumor progression, whereas HIF-1α may exert context-dependent or even opposing effects, highlighting functional divergence within the HIF family [23,24].

Beyond transcriptional control, the VHL-HIF axis is increasingly recognized as a central regulator of mitochondrial function and redox homeostasis. Persistent HIF activation under VHL deficiency suppresses mitochondrial oxidative phosphorylation, inhibits pyruvate entry into the tricarboxylic acid cycle via PDK induction, and remodels mitochondrial respiration and reactive oxygen species (ROS) dynamics, thereby promoting metabolic adaptation and oxidative stress tolerance in ccRCC [5,18]. *VHL* deficiency is associated with increased ROS, mitochondrial stress, and inflammation, potentially involving Lon protease and c-Jun N-terminal kinase signaling, and may contribute to ccRCC progression [25]. In contrast, VHL loss in ccRCC drives HIF-dependent metabolic reprogramming, whereas sirtuin 4 (SIRT4) downregulation promotes tumor progression. SIRT4 suppresses glutamine metabolism and inhibits the HIF-1α/heme oxygenase-1 axis, leading to ROS accumulation and apoptosis, particularly in VHL-deficient cells [26]. VHL deficiency promotes ROS accumulation and inflammation via an lipocalin 2 (LCN2)-dependent pathway, thereby contributing to a tumor-promoting microenvironment [27]. Collectively, VHL deficiency drives HIF-dependent metabolic reprogramming, redox imbalance, and inflammation, with key regulators, such as SIRT4 and LCN2, contributing to a tumor-promoting microenvironment in ccRCC. These findings provide mechanistic insights into VHL-driven tumor biology and may inform future studies on targeted therapeutic strategies. Collectively, VHL loss in ccRCC should be regarded not simply as an HIF stabilization trigger but also as a system-level oncogenic perturbation that integrates pseudohypoxia, proteostatic dysregulation, metabolic rewiring, redox imbalance, and immune remodeling, thereby creating convergent therapeutic vulnerabilities.

## 3. Targeting the VHL-HIF Axis: Therapeutic Vulnerabilities and Natural Product-Based Modulation

The VHL-HIF axis represents a central oncogenic dependency in ccRCC and has been therapeutically targeted by selective HIF-2α inhibitors, which suppress HIF-driven transcriptional programs in VHL-deficient tumors and consequently attenuate angiogenic, metabolic, and survival signaling [28]. Despite these advances, therapeutic efficacy in ccRCC remains limited by adaptive resistance mechanisms driven by the persistent phosphoinositide 3-kinase (PI3K)/protein kinase B (AKT)/mechanistic target of rapamycin (mTOR) pathway activation, which promotes metabolic reprogramming characterized by enhanced glycolysis and de novo lipogenesis, thereby sustaining tumor growth and survival under therapeutic pressure [29]. In contrast, ccRCC is a metabolically driven malignancy characterized by coordinated dysregulation of the VHL/HIF axis and the PI3K/AKT/mTOR pathway, which orchestrate alterations in glucose, lipid, and amino acid metabolism [30]. These interconnected signaling and metabolic networks enable tumor cells to adapt to hypoxic and nutrient-limited conditions, thereby supporting sustained proliferation, metastasis, and therapeutic resistance [14,31]. Collectively, these findings highlight that ccRCC progression is driven by integrated VHL-HIF and PI3K/AKT/mTOR signaling-metabolic networks that confer adaptive plasticity and therapeutic resistance. This underscores the need for multitarget strategies to disrupt these interconnected vulnerabilities. These observations suggest that effective therapeutic intervention in ccRCC may require the simultaneous disruption of hypoxia signaling, metabolic adaptation, and stress-response pathways rather than the blockade of a single oncogenic node. This provides a conceptual basis for evaluating selected natural compounds as potential multi-target modulators of interconnected vulnerabilities in VHL-deficient tumors.

Notably, the therapeutic challenges of ccRCC do not arise from a single dominant signaling lesion alone but from the capacity of VHL-deficient tumors to engage in compensatory angiogenic, metabolic, and immune-adaptive programs under treatment pressure. In this context, natural products may be particularly relevant not as stand-alone substitutes for current therapies but as adjunctive network modulators capable of weakening compensatory signaling, enhancing stress vulnerability, and improving therapeutic responsiveness.

## 4. Natural Products Targeting the VHL-HIF Axis, Metabolic Plasticity, and Tumor-Immune Interactions in ccRCC

To improve conceptual consistency and translational relevance, the representative natural compounds discussed in this review were selected according to several criteria: (i) reported regulatory effects on key pathogenic pathways involved in ccRCC, including the VHL-HIF axis, metabolic reprogramming, redox homeostasis, and tumor-immune interactions; (ii) evidence of multi-target or pleiotropic biological activities rather than isolated single-pathway effects; (iii) availability of experimental evidence derived from ccRCC models, RCC-related systems, or mechanistically relevant cancer studies; and (iv) frequent representation in the contemporary literature on natural products and renal cancer biology. Accordingly, compounds such as curcumin, resveratrol, quercetin, and epigallocatechin-3-gallate (EGCG) were included as representative examples illustrating distinct yet overlapping network-modulating properties, rather than as an exhaustive list of therapeutic candidates.

**Figure 2:**
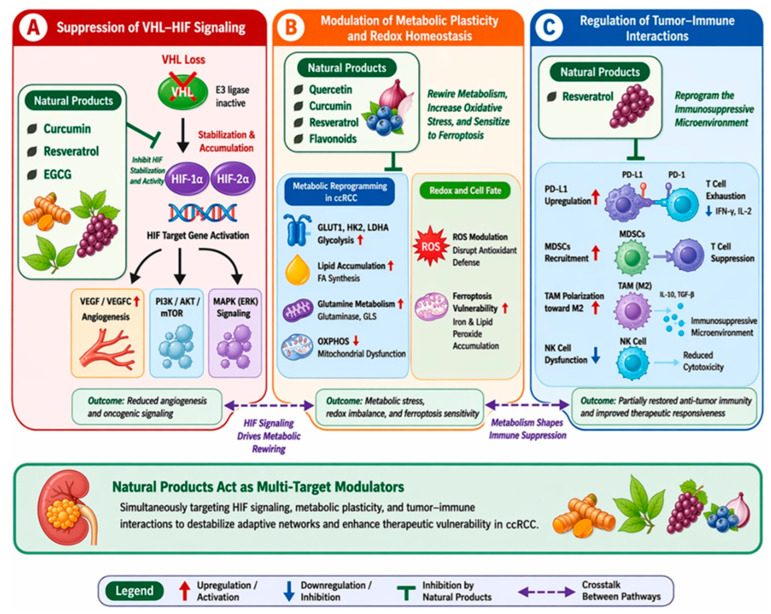
Natural products targeting the VHL-HIF axis, metabolic plasticity, and tumor-immune interactions in clear cell renal cell carcinoma. (**A**) Natural compounds such as curcumin, resveratrol, and EGCG suppress HIF signaling and angiogenesis by modulating pathways including phosphoinositide 3-kinase (PI3K)/protein kinase B (AKT)/mechanistic target of rapamycin (mTOR) and mitogen-activated protein kinase (MAPK). (**B**) Natural products interfere with metabolic reprogramming by targeting glycolysis, lipid metabolism, mitochondrial function, and redox balance, thereby promoting metabolic stress and ferroptosis susceptibility. (**C**) These agents may also modulate tumor–immune interactions by influencing immune checkpoints and macrophage polarization, although direct evidence in ccRCC remains limited. Collectively, these representative compounds illustrate the potential of natural products to function as multi-target modulators capable of perturbing interconnected oncogenic pathways in ccRCC; however, most evidence remains preclinical and context-dependent. The schematic is intended as a conceptual illustration integrating current experimental observations rather than a fully validated biological network model. The figure was generated with assistance from ChatGPT (OpenAI) version 5.2 and was subsequently reviewed, edited, annotated, and manually refined by the authors.

To delineate the mechanistic basis of natural product-mediated modulation of ccRCC, we summarized their effects across three major vulnerability axes: the VHL-HIF signaling pathway, metabolic plasticity and redox homeostasis, and tumor-immune interactions. Certain natural compounds exhibit multi-target regulatory activities that extend beyond single-pathway inhibition, enabling coordinated modulation of interconnected oncogenic processes. Specifically, these agents can attenuate HIF-driven angiogenic signaling; disrupt metabolic reprogramming by targeting glycolysis, mitochondrial function, and lipid metabolism; and influence immune remodeling by modulating immune checkpoints and macrophage polarization. Although the extent of immune reprogramming in ccRCC remains unclear, emerging evidence supports the role of the selected compounds in partially reshaping the immunosuppressive tumor microenvironment. Collectively, these findings suggest that selected natural products may interfere with adaptive oncogenic networks associated with ccRCC progression (Figure 2).

Natural products have emerged as potential multitarget modulators capable of intervening in the complex signaling and metabolic networks that define ccRCC. However, the compounds discussed in this review, including curcumin, resveratrol, quercetin, and EGCG, should be regarded as illustrative examples of pleiotropic bioactive agents rather than universally effective therapeutic candidates. Current evidence remains heterogeneous and is largely derived from preclinical studies with variable experimental systems, treatment conditions, and disease contexts [32,33,34]. Consistent with this notion, accumulating evidence indicates that natural compounds exert anticancer effects by simultaneously regulating multiple hallmarks of tumor biology, including proliferation, apoptosis, angiogenesis, and metabolic reprogramming, thereby offering broader therapeutic potential in renal cell carcinoma than previously recognized [35]. Unlike conventional targeted therapies that primarily inhibit single oncogenic nodes, some natural compounds have been reported to regulate multiple interconnected pathways, thereby offering a system-level strategy to counteract adaptive plasticity and therapeutic resistance in VHL-deficient ccRCC. This is particularly relevant, given that resistance in ccRCC is driven by a coordinated crosstalk between HIF signaling, metabolic reprogramming, and the tumor microenvironment [18], whereas natural products have been shown to simultaneously regulate multiple oncogenic processes and enhance therapeutic sensitivity [36].

### 4.1. Modulation of the VHL-HIF Axis

Natural products targeting the VHL-HIF axis in ccRCC can be broadly categorized into three functional groups: agents that suppress HIF stabilization or transcriptional activity, compounds that interfere with upstream signaling pathways that sustain hypoxia-responsive programs, and molecules that enhance sensitivity to targeted therapies by disrupting adaptive stress responses. Given the central role of the VHL-HIF axis in ccRCC pathogenesis, several natural compounds have been reported to interfere with HIF signaling and its downstream transcriptional programs [37]. Polyphenolic compounds such as curcumin and resveratrol suppress HIF-1α stabilization and downstream hypoxia-responsive signaling, partly by inhibiting upstream pathways including PI3K/AKT/mTOR and mitogen-activated protein kinase (MAPK) cascades, thereby attenuating angiogenic and metabolic gene expression. Curcumin inhibits HIF-1 activity and downregulates HIF target genes [38], while suppressing the PI3K/AKT and MAPK signaling pathways involved in tumor progression [39]. Similarly, resveratrol inhibits HIF-1α/VEGF signaling and modulates PI3K/AKT/mTOR and MAPK pathways, contributing to reduced angiogenesis and tumor growth, including renal cell carcinoma models [40]. Xu et al. reported that curcumin overcomes sunitinib resistance in ccRCC cells, likely through a disintegrin and metalloproteinase with thrombospondin motifs 18 upregulation-mediated induction of ferroptosis, thereby suppressing tumor cell proliferation [41]. Interestingly, curcumin exerts a concentration-dependent dual effect on RCC, promoting cytoprotection at low doses, inducing cell death at high doses, and acting as a key regulator mediated by the AMP-activated protein kinase (AMPK)/endoplasmic reticulum (ER) stress pathways at low concentrations and oxidative stress at high concentrations [42]. In contrast, resveratrol exerts antitumor effects in ccRCC by promoting RBM15-mediated cellular senescence, primarily through destabilization of CCNB1 mRNA and suppression of E1A binding protein p300 (EP300)/CREB-binding protein (CBP) signaling, thereby inhibiting tumor proliferation and progression both in vitro and in vivo [43]. Co-treatment with tivozanib and resveratrol enhances antitumor activity by promoting apoptosis, inhibiting proliferation and migration, and suppressing HIF-1α/VEGFC signaling, suggesting that resveratrol may improve efficacy and help reduce tivozanib-associated toxicity in kidney cancer [44]. Collectively, these findings indicate that natural compounds such as curcumin and resveratrol exert multifaceted antitumor effects in ccRCC by targeting the VHL-HIF axis and its associated signaling networks, thereby suppressing angiogenesis, metabolic reprogramming, and tumor progression, while offering potential strategies to overcome drug resistance and enhance therapeutic efficacy.

Epigallocatechin-3-gallate (EGCG) has been reported to suppress HIF-1α activity and downregulate vascular endothelial growth factor (VEGF) expression, thereby inhibiting tumor angiogenesis [45]. These effects are associated with the modulation of upstream signaling pathways, including PI3K/AKT and MAPK cascades, which regulate HIF-dependent transcriptional networks [46]. Given that ccRCC is characterized by constitutive VHL-HIF-VEGF axis activation, these findings suggest that EGCG may exert antiangiogenic effects by interfering with HIF-driven transcriptional programs. Moreover, *TP53* was identified as a key target of quercetin in ccRCC, with integrated computational and experimental analyses demonstrating that quercetin suppresses tumor cell proliferation and migration, potentially by regulating *TP53* signaling [47]. Furthermore, Sorafenib prolongs progression-free survival in patients with treatment-refractory ccRCC; however, its therapeutic benefits are constrained by toxicity and modest overall survival improvement, underscoring the need for more effective and less toxic treatment strategies [48]. Overall, these findings support the idea that natural compounds may attenuate VHL-HIF-driven tumor progression by suppressing angiogenic signaling and reshaping broader adaptive networks that sustain proliferation, metabolic flexibility, and treatment resistance in ccRCC.

### 4.2. Targeting Metabolic Reprogramming and Redox Homeostasis

Metabolic rewiring is a hallmark of ccRCC and is characterized by coordinated alterations in glucose, lipid, and amino acid metabolism [4]. Driven largely by HIF activation mediated by VHL loss, ccRCC cells exhibit enhanced glycolysis, increased glutamine dependence, suppressed mitochondrial oxidative phosphorylation, and a lipid-rich phenotype that supports biomass accumulation and stress adaptation [49]. HIF-dependent transcriptional programs further reinforce this phenotype by upregulating key glycolytic regulators, such as GLUT1, HK2, and lactate dehydrogenase A (LDHA), while repressing mitochondrial oxidative metabolism [50,51]. In parallel, aberrant lipid storage and redox buffering contribute to tumor survival under hypoxic and nutrient-limited conditions and may also create context-dependent vulnerabilities to oxidative stress and ferroptotic cell death [5,52].

Natural compounds such as curcumin, quercetin, resveratrol, and other flavonoids have been reported to interfere with glycolytic signaling, lipid metabolic pathways, and redox homeostasis across ccRCC and related cancer models, largely through multi-target modulation of metabolic enzymes and stress-response pathways [36,53,54]. Rather than acting exclusively through a single enzyme, these agents appear to modulate broader metabolic programs, including AMPK-associated stress signaling, mitochondrial function, and antioxidant balance [39,40,53,54]. These findings suggest that natural products may exploit the metabolic plasticity of ccRCC by destabilizing adaptive networks that support tumor bioenergetics, redox tolerance, and therapy resistance. Moreover, ccRCC exhibits a lipid-rich phenotype with an increased reliance on fatty acid synthesis and storage, highlighting lipid metabolism as a critical component of tumor progression [5,55]. Collectively, these findings suggest that natural compounds may interfere with metabolic vulnerabilities in ccRCC by regulating glycolytic pathways. This metabolic targeting may further sensitize ccRCC cells to oxidative stress and ferroptosis.

### 4.3. Modulation of Tumor-Immune Interactions and the Immunosuppressive Microenvironment

Besides metabolic rewiring, ccRCC is characterized by a paradoxical tumor microenvironment (TME) marked by substantial immune infiltration and profound functional immunosuppression, a phenotype largely driven by VHL loss and sustained HIF activation. Mechanistically, aberrant VHL-HIF signaling reshapes the immune landscape by promoting angiogenesis, recruiting immunosuppressive cell populations, and impairing effective antitumor immunity [56,57,58]. In ccRCC, HIF-dependent transcriptional programs extend beyond the canonical control of angiogenesis and metabolism to actively rewire the tumor immune microenvironment, drive PD-L1 expression, promote the accumulation of immunosuppressive myeloid populations, such as myeloid-derived suppressor cells (MDSCs), and skew tumor-associated macrophages toward an M2-like phenotype, thereby establishing a permissive niche for immune evasion and therapeutic resistance [6,16,18,20]. These coordinated processes foster T cell dysfunction and exhaustion, weaken effective antitumor immunity, and explain why only a subset of patients with ccRCC derive durable benefits from immune checkpoint blockade [20,59,60,61,62]. Tong et al. demonstrated that VHL mutation–driven HIF activation establishes an immunosuppressive tumor microenvironment that limits natural killer (NK) cell infiltration and function in ccRCC. Restoring VHL or inhibiting HIFα reverses these effects, enhancing NK cell-mediated anti-tumor immunity and highlighting HIF targeting as a promising therapeutic strategy [63]. Collectively, these findings indicate that VHL-HIF-driven immune remodeling establishes an immunosuppressive TME that limits effective antitumor immunity and contributes to variable responses to immune checkpoint blockade in patients with ccRCC.

In this context, natural products are increasingly recognized as pleiotropic modulators of tumor-immune interactions, capable of reshaping the tumor immune microenvironment by regulating inflammatory and redox signaling, cytokine networks, immune checkpoint pathways, and macrophage polarization, dynamically influencing tumor-associated immune responses [64,65,66,67]. Several phytochemicals have been reported to modulate tumor-associated macrophage polarization, a central determinant of immunosuppressive remodeling in cancer [68]. Regarding ccRCC, direct evidence for natural product-mediated immune remodeling remains limited. Resveratrol is currently one of the few compounds with disease-relevant experimental support, as it has been shown to affect ccRCC cell senescence, while also influencing macrophage-associated signaling [43]. Although additional evidence from other cancer models indicates that resveratrol can suppress M2-like macrophage polarization and modulate tumor-associated immune responses, these observations should be interpreted as supporting rather than definitive evidence of immune reprogramming in ccRCC [43,69,70]. Overall, natural products may influence macrophage-associated immunosuppressive remodeling in ccRCC; however, this possibility requires further validation in tumor-specific immune models and translational settings.

Although several natural compounds have demonstrated immunomodulatory effects in experimental cancer models, direct evidence supporting tumor-specific immune reprogramming in ccRCC remains limited and sometimes inconsistent across different biological systems. Moreover, many reported immune-related effects are inferred from non-ccRCC models or broader inflammatory contexts, limiting definitive conclusions regarding their therapeutic relevance in renal cancer. Therefore, the compounds discussed in this review should primarily be considered representative examples illustrating the potential of natural products to modulate tumor-immune interactions rather than definitive immunotherapeutic agents for ccRCC.

Table 1 summarizes the representative natural products and their reported molecular targets, biological effects, and potential translational relevance in ccRCC and related renal cell carcinoma models. The “ccRCC-specific evidence” column indicates whether the supporting data are derived directly from ccRCC models (Yes), limited ccRCC-specific studies (Yes, limited), partially supported by RCC-related or indirect evidence (Partial), or inferred from non-ccRCC cancer models (No). Overall, most evidence remains preclinical; however, these compounds exhibit multitarget activities across interconnected pathways, including HIF signaling, metabolic reprogramming, and redox homeostasis, supporting their potential role as network-level modulators in VHL-deficient ccRCC.

## 5. Systems-Level Integration: Natural Products as Network Modulators in VHL-Deficient ccRCC

Importantly, the framework proposed in this review should be interpreted primarily as a conceptual and hypothesis-generating model rather than a quantitatively validated mechanistic system. Although accumulating evidence supports extensive crosstalk among VHL-HIF signaling, metabolic rewiring, redox adaptation, and tumor-immune interactions, the dynamic hierarchy and causal relationships among these interconnected processes remain incompletely understood. Therefore, the current framework is intended to provide a systems-level perspective for integrating these adaptive oncogenic programs in ccRCC and for guiding future mechanistic and translational investigations. Based on the interconnected roles of VHL loss, HIF activation, metabolic rewiring, and immune suppression, ccRCC should not be conceptualized as a tumor driven by isolated signaling abnormalities; rather, it should be considered as a dynamically adaptive system governed by tightly coupled oncogenic networks [37,71]. Within this framework, the VHL-HIF axis functions as a central organizing hub that coordinates angiogenesis, metabolic plasticity, redox homeostasis, and tumor-immune interactions, thereby enabling tumor cells to continuously adapt to environmental and therapeutic stressors [5,14,21]. Notably, this system-level organization underlies the fundamental limitations of current therapeutic strategies. While targeted inhibition of individual pathways, such as VEGF signaling or immune checkpoints, has yielded clinical benefits, these approaches often fail to achieve durable responses owing to the compensatory activation of parallel pathways and adaptive rewiring of tumor-intrinsic and microenvironmental programs [6,72].

Importantly, this system-level organization underlies the fundamental limitations of current therapeutic strategies. While targeted inhibition of individual pathways, such as VEGF signaling or immune checkpoints, has yielded clinical benefits, these approaches often fail to achieve durable clinical responses owing to the compensatory activation of parallel pathways and adaptive rewiring of tumor-intrinsic and microenvironmental programs [6,8,30]. For instance, the inhibition of angiogenesis may exacerbate hypoxia and further activate HIF-dependent metabolic and immunosuppressive pathways, whereas immune checkpoint blockade may be limited by persistent myeloid-driven immunosuppression and metabolic constraints within the tumor microenvironment [6,16,18].

Natural products may provide complementary therapeutic approaches with multitarget regulatory properties. Rather than acting as single-target inhibitors, these compounds exhibit polypharmacological properties that enable the simultaneous modulation of multiple interconnected pathways, including HIF signaling, PI3K/AKT/mTOR activity, metabolic flux, oxidative stress, and immune regulation [7,32,33,34]. Such multitarget activity is particularly relevant in ccRCC, where tumor progression and therapeutic resistance arise from network-level redundancy and plasticity rather than dependency on a single oncogenic driver [8,30].

From a systems biology perspective, certain natural products may partially perturb adaptive oncogenic networks that can perturb the robustness of oncogenic circuits that sustain tumor adaptation. By concurrently attenuating angiogenic signaling, disrupting metabolic homeostasis, and partially reprogramming the immunosuppressive tumor microenvironment, these agents may lower the threshold for therapeutic vulnerability and enhance the efficacy of existing treatments [32,33,34,36]. This concept is supported by accumulating evidence that compounds such as curcumin, resveratrol, and flavonoids modulate multiple cancer hallmarks, including cell proliferation, apoptosis, redox balance, and immune cell function [35,36,53]. Moreover, the ability of natural products to influence redox homeostasis and metabolic stress responses may be particularly important in VHL-deficient tumors that operate near the limits of oxidative and metabolic tolerance [5,18]. By perturbing these adaptive buffers, natural compounds may expose latent vulnerabilities such as ferroptosis sensitivity, mitochondrial dysfunction, and immune reactivation, thereby providing opportunities for combination strategies with targeted therapy or immunotherapy [36,52]. Despite these promising mechanistic insights, several critical challenges remain unresolved. First, most of the current evidence was derived from in vitro studies or non-ccRCC-specific models, limiting the translational relevance of these findings [35,36]. Second, the pleiotropic nature of natural compounds, which is advantageous at the system level, complicates mechanistic dissection and pharmacological optimization [32,33,34]. Third, issues related to bioavailability, pharmacokinetics, and standardization continue to hinder its clinical application [7]. Therefore, future research should prioritize integrative approaches that combine multi-omics profiling, spatial transcriptomics, metabolomics, tumor microenvironment modeling, and clinically relevant systems to better define how natural products interact with VHL-HIF-driven networks in ccRCC. In particular, quantitative systems biology approaches, including computational network modeling, dynamic pathway analysis, and multi-scale data integration, may help clarify the nonlinear interactions and adaptive feedback mechanisms underlying therapeutic resistance and tumor plasticity. Moreover, the development of immunocompetent models, patient-derived organoids, and immune co-culture systems will be essential for experimentally validating these network interactions and determining the translational relevance of natural product-mediated modulation in ccRCC [6,16].

Table 2 summarizes the key system-level features of ccRCC driven by VHL loss and HIF activation, highlighting the associated molecular events, functional consequences, and limitations of current therapeutic strategies. These interconnected processes, including pseudohypoxia, angiogenesis, metabolic rewiring, redox adaptation, and immunosuppressive remodeling, form a dynamic oncogenic network that underlies tumor progression and therapeutic resistance. Natural products have been proposed as multitarget modulators that simultaneously perturb these pathways, thereby destabilizing network robustness, exposing context-dependent vulnerabilities, and enhancing the therapeutic responsiveness to ccRCC.

## 6. Conclusions and Future Perspectives

ccRCC represents a paradigmatic example of a system-driven malignancy, in which VHL loss initiates a cascade of interconnected alterations encompassing pseudohypoxia, metabolic rewiring, redox imbalance, and immune suppression [5,14,18]. These processes do not operate independently; rather, they form a self-reinforcing adaptive network that underlies tumor progression, heterogeneity, and therapeutic resistance [8,30]. Within this complex landscape, natural products have emerged as promising adjunctive agents capable of modulating multiple nodes in the VHL-HIF-metabolism-immune axis. Unlike conventional targeted therapies, which often fail due to pathway redundancy and compensatory signaling, natural compounds offer a system-level approach by simultaneously influencing angiogenesis, metabolic plasticity, oxidative stress, and tumor–immune interactions [7,32,33,34]. This multi-target capacity positions natural products not as replacements for current therapies but as network-level modulators that may enhance therapeutic responsiveness and overcome adaptive resistance [35,36]. However, the clinical translation of these findings remains at an early stage. Current evidence supporting the role of natural products in ccRCC is limited, particularly in the context of tumor-specific immune modulation and combination therapy [35,36]. Future studies should focus on validating these mechanisms in clinically relevant models, optimizing their pharmacological properties, and designing rational combination strategies with immune checkpoint inhibitors and targeted therapies [6,7].

In summary, reframing ccRCC as a systemic disease underscores the need for therapeutic strategies that extend beyond single pathway inhibition. Selected natural compounds, through their reported multi-target and network-modulating activities, may offer a promising avenue for disrupting the adaptive resilience of VHL-deficient tumors [32,33,34]. Advancing this paradigm will require rigorous mechanistic investigation and translational validation; however, it holds significant promise for improving outcomes in patients with ccRCC. A network-oriented therapeutic framework may provide additional perspectives for improving future ccRCC treatment strategies, positioning natural products as integrative modulators capable of collapsing the resilience of VHL-deficient tumor systems.

## 7. Discussion

ccRCC is increasingly recognized as a system-level malignancy rather than a tumor driven by a single genetic alteration. Loss of VHL function establishes a persistent pseudohypoxic state that links angiogenesis, metabolic rewiring, redox imbalance, and immune remodeling into an interconnected oncogenic network [73,74]. Within this framework, the VHL-HIF axis acts as a central regulator that coordinates tumor adaptation to hypoxia, nutrient limitation, and therapeutic stress [28,75]. This integrated model helps explain the strong adaptive capacity and therapeutic resistance observed in ccRCC.

Therapeutic resistance in ccRCC does not arise solely from incomplete inhibition of one pathway. Instead, resistance is driven by compensatory signaling and adaptive rewiring among interconnected pathways [8,30,31,72]. Hypoxia further amplifies HIF-dependent metabolic and immunosuppressive programs, promoting myeloid-driven immune suppression and limiting T-cell function within the tumor microenvironment [6,14,20,50,76]. These features reduce the long-term effectiveness of both anti-angiogenic therapy and immune checkpoint blockade. Collectively, these observations highlight the limitations of single-pathway therapeutic approaches in ccRCC.

Selected natural products may represent a complementary therapeutic strategy in specific experimental contexts because certain compounds have demonstrated polypharmacological properties in specific experimental contexts. Unlike conventional targeted agents that focus on a single molecular target, natural compounds can simultaneously regulate multiple pathways, including HIF signaling, PI3K/AKT/mTOR activity, metabolic reprogramming, oxidative stress, and immune modulation [32,33,34]. Such broad regulatory activity may be particularly relevant in ccRCC, where tumor progression depends on coordinated signaling and metabolic adaptation.

This pleiotropic capacity aligns well with the systems-level nature of ccRCC, in which tumor progression is driven by interconnected signaling and metabolic pathways regulated by VHL-HIF signaling [5,30,32,33,34]. Compounds such as curcumin, resveratrol, quercetin, and EGCG can simultaneously modulate angiogenic, metabolic, and immune-related pathways in ccRCC, supporting their role as multi-target regulators rather than single-pathway inhibitors. Nevertheless, most current evidence remains preclinical and requires further validation in ccRCC-specific, immunocompetent, and spatially resolved models to better define tumor–immune interactions and translational relevance [5,36,39,40,53,64,65,66]. In addition, the reported biological effects of these compounds may vary substantially depending on tumor context, experimental design, dosage, formulation, and treatment duration, highlighting the heterogeneity and context-dependent nature of current findings.

The therapeutic potential of natural products may be greatest in combination settings. From a systems biology perspective, these agents may weaken the adaptive buffering capacity of oncogenic networks and increase tumor sensitivity to existing therapies. By simultaneously disrupting redox balance, metabolic plasticity, and immune evasion, natural compounds may enhance responses to targeted therapy and immune checkpoint blockade [32,34,52]. However, these synergistic effects remain largely experimental and require further confirmation in clinically relevant models.

Despite these promising findings, several important translational challenges remain. Most available studies rely predominantly on in vitro systems or non-ccRCC-specific models, limiting direct clinical applicability and reducing the ability to fully capture the complexity of tumor–microenvironment interactions in vivo [7,35,36]. In addition, the pleiotropic nature of natural compounds, while advantageous for multi-target modulation, complicates mechanistic interpretation, pharmacological optimization, and dose standardization [32,33,34].

Importantly, many natural compounds exhibit poor aqueous solubility, limited intestinal absorption, rapid metabolic degradation, short plasma half-life, and variable pharmacokinetic profiles, all of which substantially reduce systemic bioavailability and therapeutic efficacy in vivo [7]. These pharmacological limitations remain major barriers to clinical translation, particularly in achieving reproducible therapeutic concentrations within tumor tissues. Moreover, variability in extraction procedures, purification methods, formulation strategies, and source materials may lead to inconsistent compound composition and biological activity across studies, thereby complicating reproducibility, quality control, regulatory evaluation, and clinical implementation.

Emerging approaches, including nanoformulations, liposomal delivery systems, polymer-based carriers, and structural modification strategies, may help improve compound stability, prolong circulation time, enhance tumor-targeted delivery, and increase systemic bioavailability [7,32,33,34]. Nevertheless, direct evidence for immune reprogramming by natural products in ccRCC remains limited, particularly in immune-competent and spatially resolved models that accurately reflect tumor-immune interactions [20,64,65]. Collectively, these limitations highlight the need for more integrative and clinically relevant experimental systems, including organoid-based platforms, immune co-culture models, spatial transcriptomics, and multi-omics approaches, to better define the translational feasibility and therapeutic potential of natural compounds in VHL-HIF-driven ccRCC.

Future studies should prioritize integrative and translational approaches that better capture the complexity of ccRCC biology. Advanced platforms, including multi-omics analysis, spatial transcriptomics, metabolomics, organoid systems, and immune co-culture models, may improve understanding of how natural products influence VHL-HIF-driven networks in different biological contexts [6,16]. Identification of predictive biomarkers and development of network-based therapeutic strategies may also improve patient stratification and rational combination therapy design. Importantly, the systems-level framework proposed in this review has not yet been experimentally validated as an integrated biological model. Although individual pathways involved in pseudohypoxia, metabolism, redox regulation, and immune remodeling have been independently characterized, their coordinated interactions in VHL-deficient ccRCC remain insufficiently resolved. Future studies incorporating longitudinal multi-omics analysis, spatially resolved profiling, and computational systems modeling will be necessary to quantitatively evaluate network behavior and identify critical regulatory nodes that may be therapeutically targetable. Such approaches may also help distinguish context-dependent versus conserved adaptive mechanisms across different ccRCC subtypes and therapeutic settings.

Collectively, the available evidence suggests that selected natural compounds may function as integrative modulators capable of influencing interconnected oncogenic networks in ccRCC. However, these findings should be interpreted cautiously, as most current evidence remains preclinical and context-dependent. Further mechanistic validation and translational studies are required before their therapeutic relevance can be fully established.

## Figures and Tables

**Table 1:** Natural products reported in clear cell renal cell carcinoma (ccRCC) or related RCC models: molecular targets, biological effects, and translational relevance.

Natural Product	Major Molecular Targets/Pathways	Biological Effects in ccRCC or RCC Models	Evidence Type	ccRCC-Specific Evidence	Translational Relevance	References
Curcumin	HIF-1α, PI3K/AKT/mTOR, MAPK, AMPK, ER stress	Inhibits HIF signaling and angiogenesis; modulates autophagy; induces ferroptosis; reverses drug resistance	In vitro, in vivo	Yes	Anti-angiogenic; metabolic modulation; sensitizer to targeted therapy	[38,39,40,41,42]
Resveratrol	HIF-1α/VEGFC, RBM15-CCNB1 axis, EP300/CBP, PI3K/AKT	Induces senescence; inhibits proliferation and migration; modulates macrophage-associated signaling	In vitro, in vivo	Yes (limited)	Anti-tumor; immune modulation; combination therapy potential	[40,43,44]
Quercetin	*TP53*, PI3K/AKT, oxidative stress pathways	Suppresses proliferation and migration; induces apoptosis; modulates redox balance	In vitro	Yes (limited)	Metabolic and redox regulation; adjunct therapy potential	[47]
EGCG	HIF-1α, VEGF, PI3K/AKT, MAPK	Inhibits angiogenesis via HIF-1α/VEGF suppression	In vitro, non-RCC supportive models	No (inferred)	Anti-angiogenic; potential HIF-targeting agent	[45,46]
Flavonoids (general)	Glycolytic enzymes (HK2, LDHA), AMPK, ROS pathways	Inhibit glycolysis; regulate lipid metabolism; disrupt redox homeostasis	In vitro, review-based evidence	Partial	Metabolic reprogramming modulation; broad-spectrum adjunct potential	[51,53,54]

**Table 2:** System-level vulnerabilities and therapeutic opportunities in VHL-deficient clear cell renal cell carcinoma.

Systems-Level Feature	Key Molecular Events	Functional Consequence	Limitation of Current Therapy	Potential Role of Natural Products	References
Pseudohypoxia (VHL loss/HIF activation)	Stabilization of HIF-1α/2α; activation of hypoxia-responsive transcriptional programs	Sustained angiogenesis, metabolic adaptation, and immune modulation	Targeting single nodes (e.g., HIF or VEGF) often leads to compensatory activation	Multi-target suppression of HIF signaling and downstream pathways	[3,14,28]
Angiogenic signaling	VEGF, PDGF upregulation; vascular remodeling	Enhanced tumor growth and nutrient supply	Anti-angiogenic therapy induces hypoxia-driven resistance	Concurrent inhibition of angiogenic signaling and upstream regulators	[14,15,45]
Metabolic rewiring	Glycolysis upregulation (GLUT1, HK2, LDHA); glutamine dependence; lipid accumulation; OXPHOS suppression	Enhanced bioenergetics and anabolic metabolism	Single-pathway metabolic targeting is insufficient due to plasticity	Broad modulation of metabolic pathways and stress signaling (e.g., AMPK)	[5,17,30]
Redox homeostasis	ROS accumulation; antioxidant buffering; mitochondrial adaptation	Protection from oxidative stress and cell death	Redox-targeted therapies are limited by compensatory antioxidant systems	Induction of oxidative stress and ferroptosis susceptibility	[18,52]
Immuno-suppressive micro-environment	PD-L1 upregulation; MDSCs recruitment; TAM (M2) polarization; NK dysfunction	T cell exhaustion and impaired antitumor immunity	Immune checkpoint blockade shows a heterogeneous response	Modulation of immune signaling and macrophage polarization	[6,16,63,64]
Adaptive resistance/network plasticity	Crosstalk among HIF, PI3K/AKT/mTOR, metabolic, and immune pathways	Dynamic adaptation and tumor heterogeneity	Single-target therapies fail due to redundancy and feedback loops	Multi-target, network-level modulation of interconnected pathways	[8,30,32,33,34]

## Data Availability

No new data were created or analyzed in this study. Data sharing is not applicable to this study.

## Abbreviations

The following abbreviations are used in this manuscript:

AKT	protein kinase B
AMPK	AMP-activated protein kinase
CBP	CREB-binding protein
ccRCC	clear cell renal cell carcinoma
EGCG	epigallocatechin-3-gallate
EP300	E1A binding protein p300
ER	endoplasmic reticulum
GLUT1	glucose transporter 1
HIF	hypoxia-inducible factor
HK2	hexokinase 2
IL-6	interleukin-6
JNK	c-Jun N-terminal kinase
LCN2	lipocalin 2
LDHA	lactate dehydrogenase A
MAPK	mitogen-activated protein kinase
MDSCs	myeloid-derived suppressor cells
mTOR	mechanistic target of rapamycin
NK cells	natural killer cells
OXPHOS	oxidative phosphorylation
PD-1	programmed cell death protein 1
PD-L1	programmed death-ligand 1
PDGF	platelet-derived growth factor
PI3K	phosphoinositide 3-kinase
ROS	reactive oxygen species
SIRT4	sirtuin 4
TAMs	tumor-associated macrophages
TERT	telomerase reverse transcriptase
TGF-β	transforming growth factor beta
TME	tumor microenvironment
VEGF	vascular endothelial growth factor
VHL	von Hippel-Lindau
